# Gustatory Imagery Reveals Functional Connectivity from the Prefrontal to Insular Cortices Traced with Magnetoencephalography

**DOI:** 10.1371/journal.pone.0021736

**Published:** 2011-07-08

**Authors:** Masayuki Kobayashi, Tetsuya Sasabe, Yoshihito Shigihara, Masaaki Tanaka, Yasuyoshi Watanabe

**Affiliations:** 1 Department of Pharmacology, Nihon University School of Dentistry, Chiyoda-ku, Tokyo, Japan; 2 Division of Oral and Craniomaxillofacial Research, Dental Research Center, Nihon University School of Dentistry, Chiyoda-ku, Tokyo, Japan; 3 Department of Physiology, Osaka City University Graduate School of Medicine, Abeno-ku, Osaka, Japan; 4 The 21st Century COE Program “Base to Overcome Fatigue”, Abeno-ku, Osaka, Japan; 5 Molecular Probe Dynamics Laboratory, RIKEN Center for Molecular Imaging Science, Chuo-ku, Kobe-shi, Hyogo, Japan; German Institute for Human Nutrition, Germany

## Abstract

Our experience and prejudice concerning food play an important role in modulating gustatory information processing; gustatory memory stored in the central nervous system influences gustatory information arising from the peripheral nervous system. We have elucidated the mechanism of the “top-down” modulation of taste perception in humans using functional magnetic resonance imaging (fMRI) and demonstrated that gustatory imagery is mediated by the prefrontal (PFC) and insular cortices (IC). However, the temporal order of activation of these brain regions during gustatory imagery is still an open issue. To explore the source of “top-down” signals during gustatory imagery tasks, we analyzed the temporal activation patterns of activated regions in the cerebral cortex using another non-invasive brain imaging technique, magnetoencephalography (MEG). Gustatory imagery tasks were presented by words (Letter G-V) or pictures (Picture G-V) of foods/beverages, and participants were requested to recall their taste. In the Letter G-V session, 7/9 (77.8%) participants showed activation in the IC with a latency of 401.7±34.7 ms (n = 7) from the onset of word exhibition. In 5/7 (71.4%) participants who exhibited IC activation, the PFC was activated prior to the IC at a latency of 315.2±56.5 ms (n = 5), which was significantly shorter than the latency to the IC activation. In the Picture G-V session, the IC was activated in 6/9 (66.7%) participants, and only 1/9 (11.1%) participants showed activation in the PFC. There was no significant dominance between the right and left IC or PFC during gustatory imagery. These results support those from our previous fMRI study in that the Letter G-V session rather than the Picture G-V session effectively activates the PFC and IC and strengthen the hypothesis that the PFC mediates “top-down” control of retrieving gustatory information from the storage of long-term memories and in turn activates the IC.

## Introduction

Noninvasive brain imaging techniques including positron emission tomography (PET) and functional magnetic resonance imaging (fMRI) have demonstrated that the anterior insular cortex (IC) and frontal operculum are activated by gustatory stimulation to the tongue and mediate gustatory processing in humans [1]–[9]. These findings are supported by cumulative results obtained by classical neurological observation of patients. Electrical stimulation of the IC in humans elicits gustatory sensations [10], [11]. Clinical studies in the patients with damage in the IC have shown deficits in taste recognition [12], [13]. Furthermore, Hausser-Hauw and Bancaud [14] reported a patient who had an epileptic focus in the frontal operculum and in whom epileptic activity or electrical stimulation in the focus produced a disagreeable taste.

In contrast to the cortical activities produced in response to an innate gustatory stimulus, how gustatory responses are modulated in the cerebral cortex is still an open issue. We have demonstrated that gustatory imagery tasks activate the IC and frontal operculum [15]; this result was supported by several fMRI studies [16], [17]. Selective attention to taste recruits activation in the IC and overlying operculum [18]. These findings suggest that the IC is likely to be activated not only by peripheral gustatory inputs but also by internal neural activities including “top-down” signals. A potential candidate for modulating neural activity in the IC is the prefrontal cortex (PFC), which is considered to be a center of “top-down” signals [19]. By contrasting activation sites during gustatory and visual imagery tasks using fMRI, we proposed that the middle and superior frontal gyri are likely to be the source of “top-down” signals that retrieve gustatory memories [15]. To test this hypothesis, it is critical to examine the order of activation of the PFC and IC. If the PFC is the source of the “top-down” signals, its activation should precede that of the IC. However, the temporal resolution of fMRI is not sufficient for examining the order of activation in the cerebral cortex, because these processes are thought to take place within a second [20].

To overcome the temporal limitations of fMRI, we used magnetoencephalography (MEG), which has better temporal resolution than other noninvasive brain imaging techniques, to address the following questions: (1) Can MEG detect the activation of the IC induced by gustatory imagery? (2) What is the temporal pattern of activation in the cerebral cortex during gustatory imagery tasks?

## Results

**Figure 1:**
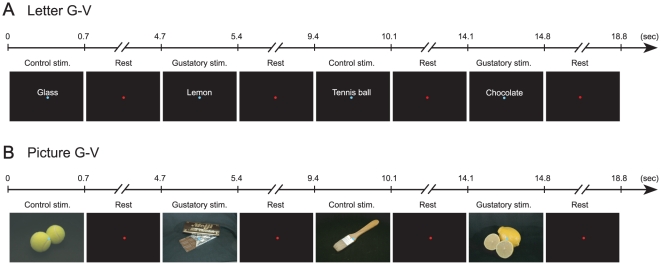
Experimental designs for the gustatory imagery tasks. ***A***. The gustatory imagery task using words (Letter G-V session). Five imagery items for each taste (sweet, salty, bitter, and sour) and non-food items were alternatively presented on the screen. Each stimulus was presented for 0.7 s, and the interstimulation interval (Rest) was set at 4 s. When the gustatory imagery item was presented (Gustatory stim.), participants were asked to recall the taste of the food; when the control (non-food) item was presented (Control stim.), participants were asked to recall its outlook. ***B***. The gustatory imagery task using pictures (Picture G-V session). Similar imagery items of food and non-food items were alternatively presented on the screen. Pictures of foods/beverages indicated that the participants should imagine tasting what was indicated, whereas non-food pictures indicated that the participants should only look at the images.

To explore regions that correlated to gustatory imagery, we designed 2 sessions of imagery tasks, Letter G-V and Picture G-V. In Letter G-V sessions, gustatory and visual imagery tasks were indicated by alternate presentation of gustatory and control words (Letter G-V, Fig. 1A). In the Picture G-V session, the gustatory and visual imagery tasks were indicated by alternate presentation of pictures on the screen (Fig. 1B). The visual analogue scale score of task achievement (see Materials and Methods) in each session was 72±3 mm (n = 9) in the Letter G-V session and 79±3 mm (n = 9) in the Picture G-V session.

### Letter G-V session

**Figure 2:**
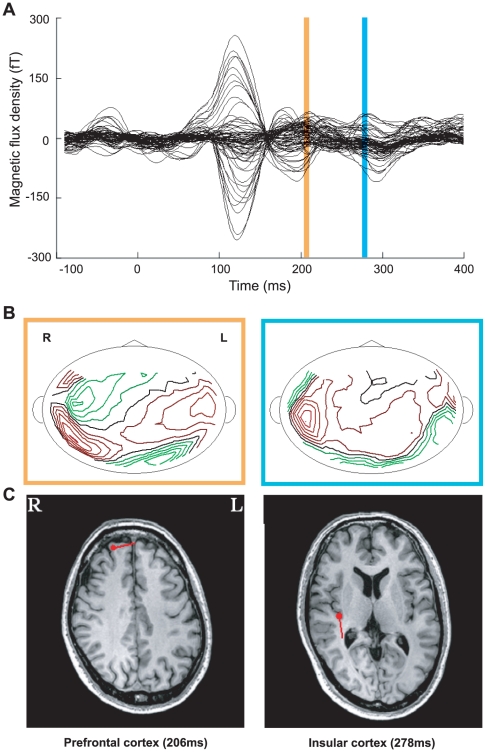
Spatiotemporal pattern of activation in the prefrontal (PFC) and insular cortices (IC). ***A***. A typical example of gustatory imagery-induced magnetic fields. ***B***. Contour maps at the time points at which the maximum dipole moment appeared in the period colored with yellow and blue strips in ***A***. There is a magnetic sink and source in the IC in each map. ***C***. Dipole localizations superimposed on magnetic resonance images at the time points at which the maximum dipole moment appeared in the period indicated by the yellow and blue strips in ***A***. In the period with the yellow strip, the dipole was located in the PFC. The horizontal direction of the dipole was left. In the period with the blue strip, the dipole was located in the IC. The horizontal direction of the dipole was posterior. R and L in panels B and C indicate right and left, respectively.

In the Letter G-V session, prominent responses were invariably observed in a part of the occipital cortex (Fig. 2). The sensor that showed the strongest activation was located in the occipital cortex; its mean latency from the onset of task presentation was 98.2±3.4 ms (n = 9).

Gustatory imagery tasks activated the IC in 7/9 participants (77.8%) in the Letter G-V session. The latency of activation was widely distributed; the latency range was 278–694 ms (mean latency: 401.7±34.7 ms, n = 7). In contrast to the gustatory imagery tasks, the visual imagery tasks in the Letter G-V session did not activate the IC (n = 9).

In 5/7 (71.4%) participants, activation in the PFC preceded the onset of activation in the IC. The latency of activation was also widely distributed (196–506 ms); however, the latency of activation in the PFC was shorter than that in the IC in all 5 participants. The mean latency in the PFC was 315.2±56.5 ms (n = 5), which was significantly smaller than that in the IC (*P*<0.05, paired *t*-test). The right PFC was activated in 3/9 participants (33.3%) in visual imagery tasks in the Letter G-V session.

### Picture G-V session

As in the case of the Letter G-V session, the Picture G-V session also invariably activated a part of the occipital cortex. The mean latency at the sensor that showed the strongest activation in the occipital cortex was 99.6±5.2 ms (n = 9) from the onset of task presentation.

In Picture G-V session, 6/9 participants (66.7%) exhibited the IC activation responding to gustatory imagery tasks. The latency of activation was widely distributed; the latency range was 312–532 ms and the mean latency was 420.3±31.7 ms (n = 6). The PFC was activated in only 1/9 participants (11.1%). The latency of activation was 490 ms.

### Correlation analysis

Preceding activation in the right PFC followed by activation in the right IC supports the hypothesis that information processing during gustatory imagery flows from the PFC to the IC as we proposed previously [15]. If this is the case, the magnitude of activation in these regions would be correlated because activity in the PFC elicits activation in the IC, which is situated downstream in the flow of information processing. To examine this possibility, we performed correlation analysis between the amplitudes of activation in the PFC and IC in the Letter G-V session.

**Figure 3:**
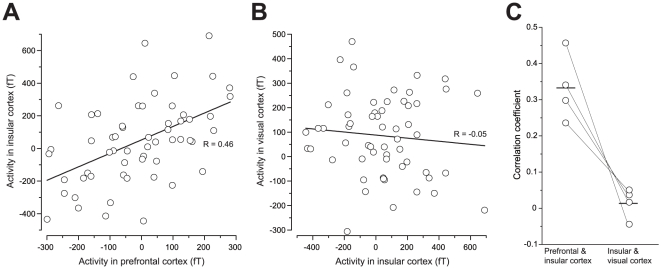
Correlation of the intensity of activation between the IC and other cortices. ***A***. An example of the correlation between the IC and the PFC in the Letter G-V session. A significant correlation was observed (R = 0.46, *P*<0.001, Pearson's correlation coefficient test). ***B***. An example of the correlation between the IC and the visual cortex in the Letter G-V session of the same participant as shown in ***A***. No significant correlation was observed (R = −0.05, *P*>0.1, Pearson's correlation coefficient test). ***C***. Summary of correlation coefficients in four participants who exhibited the activation in the PFC and IC in the right hemisphere in the Letter G-V session.

We analyzed 4 participants who showed activation in the PFC followed by activation in the right IC as described above. The amplitude of activation in the PFC and IC in each trial was measured at the peak of the averaged traces. The sensors of interests (SOIs) were those in which the largest amplitudes of activation in the PFC, IC and primary visual cortex were observed. Figure 3 shows a typical example of a correlation between the amplitude of the SOIs in the PFC and IC. The mean value of correlation value (R) was 0.33±0.01 (n = 4, *P*<0.0001, Pearson's correlation coefficient test). In the same participants, no significant correlation was seen between the amplitudes of the SOIs in the visual cortex and the PFC/IC (Fig. 3B; 0.01±0.002; n = 4). There was a significant difference in the R value between the PFC vs. the IC and the visual cortex vs. the IC (Fig. 3C; *P*<0.02, paired *t*-test).

### Laterality of activation sites during gustatory imagery

In the Letter G-V session, the bilateral IC was activated in 2/7 participants, the right IC in 4/7 participants and left IC in 1/7 participants. In the Picture G-V session, only the right hemisphere was activated in all of the participants who showed activation in the IC (6/9). To examine whether activation in the right IC was dominant for gustatory imagery, a Fisher's exact test was performed (see Materials and Methods). The *P* value was more than 0.12: we therefore concluded that there is no significant dominance between the right and left IC during gustatory imagery.

In terms of PFC activation in the Letter G-V session, 4 participants showed the right PFC activation, while one participant showed bilateral PFC activation. In the Picture G-V session, the right hemisphere was activated in one participant who showed activation in the PFC (1/9). Similar to IC activation, a Fisher's exact test revealed that there was no significant dominance between the right and left PFC during gustatory imagery (*P*>0.8, Fisher's exact test).

## Discussion

In the present study, we investigated the functional connection of brain regions during gustatory imagery with MEG. We identified magnetic responses in the occipital cortex, the IC, and the PFC. The magnetic response in the occipital cortex could be regarded as a visual response, based on the visual stimuli we used, the goodness of fit value, the localization, and reaction time as shown previously [21]. Gustatory imagery directed by word presentation induced activation in the IC that was preceded by activation of the PFC in 5/9 participants. There was a significant correlation between the amplitudes of activities in the PFC and IC, whereas no significant correlation was observed between the IC and visual cortex. These lines of evidence support our hypothesis that the source of “top-down” signals is likely to be in the PFC and that these signals affect neural activity in the IC.

### Technical consideration

Non-invasive brain imaging techniques have intensively promoted studies in human brain anatomy and physiological functions. Among these brain imaging techniques, fMRI and PET are considered to have superior spatial resolution. The standard values of the minimum spatial resolution are ∼1–2 mm in fMRI and 2–4 mm in PET [22]. In contrast, MEG, which is used in the present study, has a spatial resolution that is ∼2 times larger than that of fMRI [23]. In addition to better spatial resolution, fMRI and PET have the advantage of being able to image deeper brain regions such as the thalamus and basal ganglia, the activities of which cannot be measured by MEG. Nevertheless, we chose MEG in the present study, because MEG is the only technology that pairs superior temporal resolution with a reasonable spatial resolution. Although electroencephalography (EEG) is another non-invasive technique for measuring brain activity with high temporal resolution, EEG was not suitable for this study because of its much lower spatial resolution. Compared to EEG, MEG has the following advantages: (1) in contrast to the electrical field, the magnetic field is hardly affected by intervening tissues such as the skull, and (2) MEG can perform measurements from more sensors than EEG. The temporal resolution of MEG is ≥1 kHz, which is much higher than that of fMRI (∼1 Hz) and enough to discriminate ms-order temporal differences. Temporal analysis of activated brain regions could be a valuable tool with which to examine which regions play a role in a certain type of information processing because information processing in the cerebral cortex is considered to at least in part be hierarchically performed in sequential order [24]. We believe that gustatory imagery tasks require several steps because our previous study demonstrated that they activate at least three regions, i.e. the IC, the PFC, and the orbitofrontal cortex (OFC) [15]. The present study aimed to examine our hypothesis that the PFC is the source sending “top-down” signals to the IC in which gustatory hallucination occurs; therefore, MEG was the most suitable technique for our purpose.

Correlation analysis may support the exploration of functional connections and help to avoid situations in which failure trials weaken signals and signal is concealed by noise. Even in a result obtained from a single participant that is composed of 2 sessions (140 trials of 70 food and 70 non-food stimuli), there is variation in imagery task performance in each stimulus response. In a failure trial in gustatory imagery, corresponding brain regions would not be activated; in turn, brain regions downstream of the sequence would also not be activated. In contrast, in a success trial in which strong gustatory hallucination occurred, the primary gustatory cortex (i.e. the IC) would show larger signals that would be induced by more intense “top-down” signals.

### The role of the IC during imagery tasks

The IC receives multimodal sensory inputs including gustation, visceral information, thermal sensation and pain via the sensory thalamus [25], [26]. Gustatory inputs via the ventroposteromedial parvicellular thalamic nucleus are conveyed to the IC in primates [27], [28]. Despite its designation, the gustatory area in the IC involves a relatively small percentage (<10%) of gustatory neurons: the majority of the population responds to jaw and mouth movements [29], [30]. However, it is worth noting that the IC has dense reciprocal connections with the amygdala, in which ∼7% of neurons respond to gustatory stimuli [31]. In addition, some gustatory information is sent to the OFC; designated as the secondary gustatory cortex, the OFC is believed to process the identification of food, satiety and food preference [32], [33]. Similar to the amygdala, the OFC also has dense reciprocal connections with the IC [34]. These networks likely drive and modulate gustatory information arising from the peripheral and, in part, the central nervous system.

According to the anatomical and physiological bases of the IC, it is likely that gustatory hallucination induces IC activation during gustatory imagery tasks. This hypothesis is supported by previous fMRI studies that demonstrated IC activation with gustatory imagery tasks [15]–[17]. In addition, a clinical report of a patient with an epileptic focus in the IC who perceived a disagreeable taste with epileptic activity or electrical stimulation in the focus [14] indicate that the IC could internally generate gustatory sensation without an external stimulus. Although our previous fMRI study using gustatory and visual imagery tasks indicated that the imagery task itself did not activate the IC [15], unknown factors might be included in the activation of the IC. Recent findings obtained with human brain imaging suggest that other information processing may activate the IC. Damasio [35] proposed that the IC is crucial for subjective emotional feeling. It has been reported that the IC is involved in attention, reasoning, planning and decision-making process relating to smoking and drug abuse [25], [36]. These higher brain functions may be included in gustatory imagery tasks. Taken together with a report that neural responses to gustatory stimuli in the IC are greater in the satiety condition [37], the IC may integrate gustatory information with physiological states by regulating sensitivity to food taste.

### The role of the PFC during imagery tasks

Imagery is one of the best-documented higher cognitive functions [38] and seems to be suitable for exploring “top-down” signals. Neural activity in early sensory areas is modified by mental states including attention and relevant prior knowledge, and the PFC is one of the sources of such modifications [39]; e.g., the PFC is activated during visual [40], [41] and auditory imagery [42]. Our previous studies also reported that the PFC is activated by gustatory imagery tasks [15], suggesting that the PFC is likely to play an essential role in generating imagery without the specificity of sensory modality. We believe that the PFC plays a crucial role in modulating gustatory information processing by “top-down” signals that are derived from gustatory memory obtained by experience. The present temporal analysis results indicate that activation of the PFC precedes IC activation, and the correlation coefficient values of the magnetic response demonstrate the high functional connectivity between these brain regions. Using functional near-infrared spectroscopy, Okamoto et al, [43], [44] have reported that the PFC is activated during gustatory memory encoding, suggesting a functional contribution for the PFC during gustatory imagery. Furthermore, our hypothesis is supported by anatomical evidence for mutual connections between the PFC and the IC [34]. The PFC is believed to play critical roles in other complex and executive functions including reasoning and planning [45]–[47]: these functions could be involved in the present experimental tasks.

In comparison to stimulation of the peripheral nervous system through methods such as sensory stimulation, it is difficult to elicit constant neural activity in the brain during imagery tasks because mental situation such as attention affect the efficacy of imagery. Furthermore, multi-step processes to generate imagery may raise the failure probability of imagery. Therefore, it is reasonable that the evoked amplitudes and latencies of signals were relatively variable in the PFC and IC.

We must acknowledge that correlation analysis was performed on 4/9 participants and therefore cannot deny that another mechanism might play a role in IC activation during gustatory imagery tasks. Although there is a possibility that the activation amplitude is under the level of statistical significance, it is also possible that subcortical structures or the OFC may contribute to IC activation, which cannot be actually detected by MEG. Indeed, our previous fMRI study reported that OFC activation during gustatory imagery task [15]. This possibility should be explored in the future.

### Laterality of the activation sites during gustatory imagery

Our previous fMRI study has revealed that the left IC is activated by gustatory imagery [15]. However, the present results revealed that there was no dominance between the right and left IC during gustatory imagery. There are several possibilities to explain this discrepancy. First, this discrepancy might be caused by the differences in what MEG and fMRI detected as signals. MEG detects magnetic fields that are directly elicited by electrical currents in mainly apical dendrites of cortical pyramidal neurons [48]. In contrast, the fMRI signal is induced by blood-oxygen dependent (BOLD) signals that are secondarily induced by neural activity. The latencies of the MEG and fMRI signals are therefore totally different: MEG signals are simultaneously induced by electrical excitation, whereas BOLD signals reach their peak ∼7 s after electrical signals. This temporal gap may reveal the temporal processing mechanism during gustatory imagery; e.g., the gustatory hallucination may first occur in the right IC, and these activities then propagate through the corpus callosum to the left IC with some modification. Pritchard et al, [13] reported that damage to the right IC produced deficits of taste recognition ipsilaterally whereas damage to the left IC caused bilateral taste deficits, suggesting that taste information from both sides of the tongue passes through the left hemisphere. Our fMRI study showed that passive gustatory perception activates the IC with the right IC dominant, whereas gustatory imagery evoked responses mainly in the left IC. Gustatory information processing is performed asymmetrically in the IC. The discrepancy in laterality between our past and present studies could also be due to the wide variation in laterality of IC activation among participants. MEG detects activation sites in individual participants, whereas our fMRI study was performed by group analysis. The difference in task presentation methods may also affect the laterality of the activation site; there was a single presentation of stimulus in the MEG experiment versus repeated stimulus presentation (4 times per one task presentation) in the fMRI study.

## Materials and Methods

### Participants

Nine volunteers with no neurological complications participated in this study (8 males and 1 female, 20 to 41 years old, mean age 30.3 years old, all right-handed). All participants gave written informed consent, and the experiment was approved by the Ethics Committee at Osaka City University, and all participants gave written informed consent for the study.

### Gustatory imagery tasks

Imagery items for gustatory imagery tasks were selected as previously described [15]. Briefly, 5 imagery items were selected for each taste: sweet, salty, bitter, and sour. Words and pictures of foods were used as imagery items. Words and pictures of non-foods were used as control items. Imagery and control items were projected onto a video screen placed 30 cm in front of the participant's eyes using a video projector (PG-B10S; SHARP, Osaka, Japan).

The examination was composed of two sessions: Letter G-V and Picture G-V (Fig. 1). In the Letter G-V session, words of foods/non-foods were alternately presented on the screen. In the Picture G-V session, pictures of foods/non-foods were alternately presented on the screen. When gustatory imagery items were presented, participants were asked to recall their taste; when control items were presented, participants were asked to recall their outlook. The stimulation period and the interstimulation interval (ISI) were 700 ms and 4000 ms, respectively, in both sessions. To reduce the noise caused by eye movement, a fixation point was presented in the center of screen; imagery and control items were presented with a blue fixation point, and a red fixation point was presented during the ISI (Rest in Fig. 1). Each session was composed of 70 stimuli: i.e., 35 food stimuli and 35 non-food stimuli. Participants had a break between the two sessions to reduce the influence of habituation and fatigue. Evoked magnetic field data measured in the two sessions were bound offline and analyzed as one evoked magnetic field dataset that had 70 food stimuli and 70 non-food stimuli. The participants were asked to subjectively rate their level of task achievement for imagery on a visual analogue scale score from 0 (minimum) to 100 (maximum) just after each task session.

### MEG measurement protocol

MEG measurement was performed in a magnetically shielded room at Osaka City University Hospital using a 160-channel helmet-type MEG system (Yokogawa Electric Corporation, Tokyo, Japan) with a magnetic field resolution of 4 fT/Hz^1/2^ in the white noise region. The sensing and reference coils in this system are both 15.5 mm in diameter with a 50-mm baseline and 23 mm of separation between each pair of sensing coils. The sampling rate was 500 Hz with a 1 to 200 Hz band-pass filter.

Participants were positioned in a supine position with the use of a horizontal-type dewar, and the MEG signal data for each stimulation period were averaged offline after analog-to-digital conversion with a low-pass 30 Hz filter. The mean magnetic signal of the pre-stimulus time period (−500 to 0 ms) was subtracted from that of the stimulus period in each channel to remove the baseline shift of the MEG data. We first assessed evoked magnetic responses in the time course of the magnetic flux density (Fig. 2A), and the distribution pattern of the magnetic field is shown on the contour maps (Fig. 1B). We then identified dipole patterns selected from at least 20 sensors and estimated an equivalent current dipole (ECD) by using software (MEG 160; Yokogawa Electric Corporation). Finally, we checked the validity of the ECD based on the location in the gray matter (Fig. 2B), the goodness of fit value (more than 80%), and the duration of the evoked response (continued for at least a few ms). The reaction time was defined as the time when the goodness of fit value for each ECD reached the local maximum level after the start of the stimulation [49].

In addition, to confirm the validity of our MEG study, somatosensory evoked field (SEFs) potentials were obtained after all stimulation sessions had been completed. SEFs were obtained by applying electrical stimulation in 0.1-ms pulses to the unilateral median nerve in the wrist using a proximally placed cathode. A forceps-like apparatus was used to fix the electrodes to the wrist to apply maximum stimulation to the median nerve. Two hundred stimuli were presented at a rate of 2 Hz, with responses passing through a 3 to 500 Hz band-pass filter with a sampling rate of 2000 Hz. The first main component of the SEFs, with a latency of approximately 20 ms, was considered as N20m.

### Magnetic resonance imaging overlay

Anatomical MRI was performed for all participants using a Philips Achieva 3.0T (Royal Philips Electronics, Eindhoven, Netherlands) to permit registration of dipole source locations with their respective anatomical locations. Before MRI scanning, five adhesive markers (Medtronic Surgical Navigation Technologies, Broomfield, USA) were attached to the skin of the participant's head (the first and second markers were located 10 mm in front of the left tragus and right tragus, the third at 35 mm above the nasion, and the fourth and fifth 40 mm right and left of the third marker). The MEG data were superimposed on the MR image using information obtained from these markers and the MEG localization coils.

### Statistical analysis

Data were expressed as the mean ± S.E.M. Paired *t*-test was used for statistical comparison of the latency and amplitude of signals. Pearson's correlation coefficient test was used for evaluation of the relationship between the amplitudes of the SOIs in the PFC, IC, and visual cortex. A Fisher's exact test was performed for a comparison of dominance between the right and left IC or PFC for gustatory imagery. In this statistical analysis, the results groups were classified into two categories for each session: (1) activated in the right IC/PFC only, and (2) activated in the bilateral or only left IC/PFC. The level of *P*<0.05 was considered to be significant.
